# Coupling coordination analysis of the economic-social-infrastructure-ecological resilience system: A case study of Zhejiang Province

**DOI:** 10.1371/journal.pone.0323673

**Published:** 2025-05-21

**Authors:** Menglin Zhang, Yuchen Hu, Yihua Mao, Jiaqi Yang

**Affiliations:** 1 College of civil Engineering and Architecture, Zhejiang University, Hangzhou, China; 2 Center for Balance Architecture, Zhejiang University, Hangzhou, China; 3 School of Digital Economics and Management, Wuxi University, Wuxi, China; 4 The Architectural Design & Research Institute of Zhejiang University Co., Ltd., Hangzhou, China; 5 Binhai Industrial Technology Research Institute of Zhejiang University, Tianjin, China,; 6 Department of Hydraulic Engineering, Tsinghua University, Beijing, China; Zhongnan University of Economics and Law, CHINA

## Abstract

Nowadays, with the acceleration of urbanization and the profound impact of climate change, urban resilience has become a key issue for sustainable development. Through the panel data of Zhejiang Province from 2014 to 2022, this study employs the coupling coordination degree (CCD) model to analyze the coupling coordination relationship of the Economic-Social-Infrastructure-Ecological resilience (ESIE) quaternary system and binary systems. The impact of the CCD of each binary system on the overall CCD is determined by the grey correlation model considering alongside the current state of the coupling coordination development to identify the key binary system that promotes the development of the overall coordination. It finds that the CCD of the ESIE quaternary system in Zhejiang’s cities exhibits a consistent upward trend, and spatially, a pattern of coordinated regional urban development centered on the city of Hangzhou and Ningbo emerges. The CCD analysis of binary systems reveals a low level of pairwise CCD between economic, infrastructure and social resilience, with prominent contradictions between the three. Additionally, the CCD of the economic-infrastructure resilience binary system is the core issue currently faced by the overall coordinated development of the ESIE quaternary system. This research not only provides a new perspective for understanding the intrinsic mechanism of coupled and coordinated development of urban resilience but also offers a scientific basis for crafting sustainable urban policies.

## 1 Introduction

In the current global context, the process of urbanization continues unabated, leading to increasingly dense urban spaces and escalating population concentrations, resulting in an unprecedented scale and complexity of urban systems [1]. Simultaneously, as global temperatures rise, natural disasters such as heatwaves, floods, and droughts are becoming more frequent. And risks such as infectious diseases, sudden social upheavals, and major security incidents are on the rise. These factors are significantly impacting urban development. Particularly in the wake of the 2020 COVID-19 pandemic, deficiencies in urban emergency management and public safety have been glaringly exposed [2], thereby highlighting the vulnerability of cities to various disasters. In this context, the concept of “resilient cities” has gained widespread attention. The construction of resilient cities aims to ensure that cities can continue to provide essential services, guarantee the quality of life and safety of citizens, and promote the long-term prosperity and stability of cities in the face of challenges. Since the interaction among multiple dimensions such as economy, society, infrastructure and ecology will affect the quantification of urban resilience [3], exploring the interaction among subsystems of urban resilience is essential for accelerating the development of resilient cities and realizing sustainable development [4].

The word “resilience” is derived from the Latin word “resilio”, indicating the ability to return to the original state [5]. In 1973, Canadian ecologist Holling [6] introduced the concept of resilience into the field of ecology for the first time in his research, defining it as “the ability of a system to absorb change, sustain and restore balance after a temporary shock”. Since then, the concept of resilience has been widely concerned and applied in the academic community. It has undergone two important evolutions from engineering resilience to ecological resilience and then to evolutionary resilience [7], with its connotations deepening continuously and its application fields expanding. At present, many well-known research institutions have given their own definitions of resilience. The United Nations International Strategy for Disaster Reduction (UNISDR) defines resilience as “the ability of a system, community or society to resist, absorb, adapt to, and recover from the effects of hazards in a timely and effective manner” [8]. The Resilience Alliance has proposed three essential characteristics of resilience: the ability to buffer disturbance, the ability to self-organize, and the ability to learn [9]. In the 21st century, resilience theory has been widely used in urban system research. In 2002, the International Council for Local Environmental Initiatives (ICLEI) advocated for the “urban resilience” agenda at the United Nations summit and incorporated it into the scope of urban development research [10], marking a new phase in the integration of resilience concepts in urban management and planning. Subsequently, many scholars began to define and discuss the concept of urban resilience based on their respective disciplinary backgrounds. Wilbanks et al. [11] defined urban resilience as the ability of urban system to prepare for, respond to, and recover from specific multiple threats, minimizing their impact on public safety, health, and the economy. Bruneau [12] proposed the “TOSE” framework, further enriching the connotation of urban resilience. This framework posits that resilience is composed of four interrelated elements: technical resilience, organizational resilience, social resilience, and economic resilience. Although different scholars have different focuses on the understanding of urban resilience, the general consensus is that the study of urban resilience is gradually emphasizing its systematization, comprehensiveness, and diversity of components.

Scientific assessment of resilience is conducive to accurately grasp the direction of urban planning, and provides an important reference for decision makers to manage cities. Therefore, many scholars are committed to the study of urban resilience evaluation, and generally construct evaluation indicator systems based on multiple dimensions such as economy, society, infrastructure and ecology. For example, Cutter et al. [13] constructed a local resilience model including dimensions of ecological, infrastructural, economic, social, institutional and community capacity, providing an improved approach for assessing local or community resilience. Jha et al. [14] introduced an urban resilience assessment system that integrates infrastructure, institutions, economy, and society. Porfiriev et al. [15] suggested constructing an indicator system from six perspectives: natural, human, social, economic, political and capital. Sun et al. [16] constructed an evaluation system from four aspects: ecological environment, economic development, social development and municipal facilities, and empirically analyzed the spatial differentiation of urban resilience in the Yangtze River Delta urban agglomeration in 2014. In general, the current research on urban resilience assessment presents the characteristics of multi-discipline, multi-scale and multi-system. According to Cutter’s review analysis [13], it is feasible to integrate attributes and capabilities of different dimensions into a comprehensive framework. Through literature research [17–21], it is found that economic resilience, social resilience, infrastructure resilience and ecological resilience are the four core dimensions of current research on urban resilience. This study further simplifies the dimensions of overlapping meanings in Cutter’s comprehensive framework, and finally selects four dimensions of economic resilience, social resilience, infrastructure resilience and ecological resilience to construct an urban resilience evaluation indicator system. Based on this, this paper defines urban resilience as follows: Urban resilience refers to the ability of a complex urban system, which is coupled by the economy, society, infrastructure, and ecology, to maintain its basic functions, structure, and system characteristics unchanged when facing uncertain shocks and disturbances [11–13,22]. It demonstrates the capacity to resist risks, allocate resources, recover and adjust, as well as innovate and learn. Such urban resilience system is also called the Economic-Social-Infrastructure-Ecological resilience (ESIE) quaternary system in this study.

Systems theory posits that systems are universally present, and within a system, several subsystems interact and mutually restrict each other, collectively influencing the system as a whole [23]. The ESIE quaternary system is inherently complex and multi-dimensional, which makes it one-sided to enhance urban resilience merely focusing on individual subsystems while neglecting the in-depth exploration of their interactions. Therefore, adopting a systems theory approach is necessary for understanding the interactions and trade-offs within urban processes, and achieving a comprehensive and in-depth understanding of the urban resilience system. Specifically, economic resilience (EnR), social resilience (SR), infrastructure resilience (IR), and ecological resilience (ElR) are all subsystems within the ESIE quaternary system. The subsystems promote and correlate each other to form a benign interactive coupling and coordination relationship, which is conducive to the overall enhancement of resilience levels [24].

**Fig 1:**
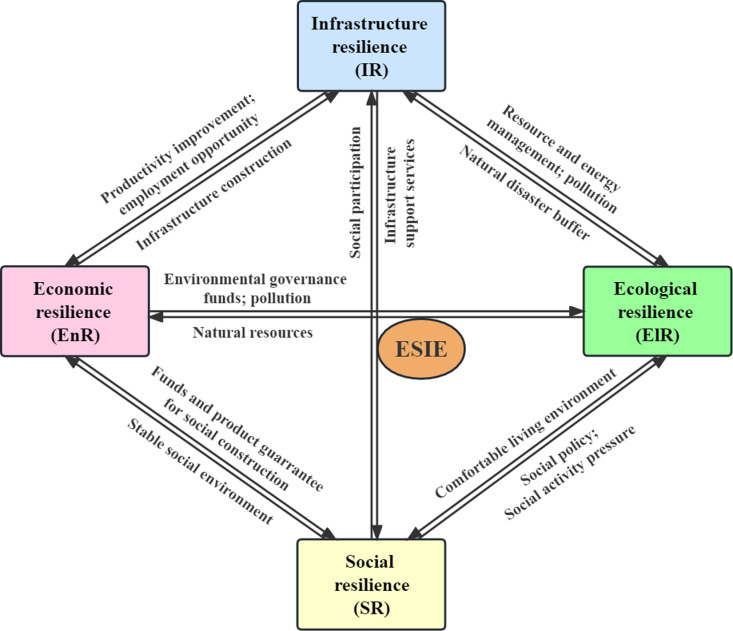
The interactions between the four subsystems of the ESIE quaternary system.

In the ESIE quaternary system, EnR, SR, IR and ElR subsystems are closely connected through a series of dynamic feedback mechanisms. They ensure the efficient and benign operation of the entire system through the circulation and conversion of matter, energy and information. In this complex system, human beings play a crucial role as agents. Human activities, such as decision-making, planning, construction, and consumption, not only directly determine the development track and evolution direction of cities, but also become a powerful driving force for the interaction between subsystems, playing a key role in maintaining the stability of the ESIE quaternary system and promoting its sustainable development [25]. The interactions between the four subsystems of the ESIE quaternary system are shown in the Fig 1. (1) The EnR subsystem is the core of the ESIE quaternary system. Various economic activities of human beings directly affect the allocation of social resources in infrastructure construction and ecological environment management, thus establishing a close relationship between EnR and other subsystems. EnR focuses on dealing with economic uncertainties. It effectively promotes the stable operation and improvement of the SR, IR and ElR subsystems by providing financial and material support for social construction, pollution control and infrastructure construction. At the same time, with the improvement of economic development level, resource consumption accelerates and pollutant production increases, which leads to the aggravation of environmental pollution. Therefore, EnR will also have a certain negative impact on ElR. (2) The SR subsystem focuses on reducing the negative impact of economic, political and demographic issues. It provides a stable social environment for EnR and the necessary social support and participation for IR by maintaining the labor market and social order. At the same time, social activities in the SR subsystem, including environmental protection policies, excessive consumption of resources, pollution emissions, etc., will have positive or negative impacts on ElR. High SR can effectively ensure urban stability and security, promote urban development [26]. (3) The IR subsystem is the foundation of the ESIE quaternary system, reflecting the hard capacity of urban operation. As the material support of the city’s disaster prevention capability, the IR subsystem covers key areas such as transportation, medical care and communication. The subsystem has resilience characteristics such as robustness and redundancy, which not only provides guarantee for the high quality of human life in daily life, but also plays the function of early warning, evacuation and resettlement when suffering disasters. IR provides guarantee for the functional realization of EnR, SR and ElR subsystems by providing sufficient and diversified infrastructure and sufficient emergency resources. Relevant studies show that infrastructure can exert its due environmental and economic benefits in regional development, and plays an important supporting role in the stable operation of the ESIE quaternary system [27,28]. In particular, a sound infrastructure can improve productivity, create employment opportunities, meet the basic needs of residents, efficiently manage resources and energy, and improve the ecological environment, thereby realizing multiple values at the economic, social and ecological levels. (4) The ElR subsystem is the safeguard of the ESIE quaternary system and it focuses on solving environmental problems and ensuring that the system has the necessary adaptability in the face of various disturbances. The ElR subsystem provides natural resources and environmental support to other subsystems while inevitably enduring the constant stress imposed by human activities, especially in the fields of industry, livelihoods and infrastructure development that rely on natural resources. For instance, in the process of urbanization, the expansion of buildings, road networks, and other urban infrastructure leads to the ongoing loss of natural habitats and the fragmentation of ecosystems [29]. As the carrier of human life, the ecological environment bears the dual impacts of human economic, social, and infrastructure development activities. On one hand, these activities exacerbate the burden on the self-regulating capacity of the ecological environment; On the other hand, through policy formulation and environmental governance investments, humans can effectively intervene and mitigate the deterioration of the ecological environment. To sum up, the elements of the ESIE quaternary system are not independent of each other, but interrelated and mutually restricted. The moderate and coordinated balance of the development of each subsystem is the basis of healthy development of the ESIE quaternary system [30]. Therefore, it is very important to study the coupling and coordination of the ESIE quaternary system to guide the construction of resilient cities.

Coupling coordination theory is a composite concept composed of two core elements, “coupling” and “coordination.” Deriving from the field of physics, the term “coupling” is used to describe the transfer of energy and interaction between circuit components, or the integrated phenomena formed by the interaction of different internal mechanisms [31,32]. In systems science, “system coupling” is commonly described as the process where two or more closely connected subsystems, through the cyclic interaction of elements such as energy, matter, and information, ultimately form a tightly integrated structural-functional entity. In this process, when the interactions and feedback mechanisms among subsystems can effectively reconcile potential conflicts and contradictions, allowing the system to achieve a state of balance, it is termed “coordination” [33]. Systems in this state exhibit a high degree of order and adaptability, capable of effectively responding to external pressures and internal changes [34]. The coupling coordination degree (CCD) is the measure of the whole dynamic development process. With further research, coupling coordination theory has gradually expanded from natural sciences to humanities and social sciences such as economics and geography. Scholars often use the CCD model to study the coupling coordination relationships between different systems, such as tourism-public services [35], natural resources-financial development-ecological efficiency [36], population-land-economic urbanization [37], digital economy-carbon emission efficiency [38], etc.

In the field of urban resilience research, CCD model is also increasingly being applied. For instance, Luo et al. [39] focused on the CCD between urban resilience and land development intensity using the panel data of Yangtze River Delta urban agglomeration. Wang et al. [40] constructed a “Size-Density-Morphology” framework to assess urban ecological resilience and measured the CCD between ecological resilience and urbanization in the Pearl River Delta. Shi et al. [41] measured the urban resilience and industrial structure in 30 provinces of China from 2011 to 2020 and analyzed the coupling coordination relationship between them. However, these studies tend to focus on the coupling and coordination between urban resilience and a single external system, with less attention paid to the coupling coordination mechanisms among subsystems within the urban resilience system. Even though some related work has been conducted, only the economic, social, and ecological resilience subsystems are primarily concentrated on. For example, Han et al. [42] utilized the revised CCD model to study the interactions among urban social, economic, and ecological resilience of the Chengdu-Chongqing urban agglomeration. Sun et al. [43] explored the coupling coordination relationship among the three subsystems of economic, social, and ecological resilience based on the analysis of the resilience of the Beijing-Tianjin-Hebei region from 2009 to 2019. Nevertheless, existing research gives less attention to infrastructure resilience. Therefore, future research should consider all dimensions of the urban resilience system more comprehensively and delve deeper into the coupling and coordination mechanisms among all subsystems.

**Fig 2:**
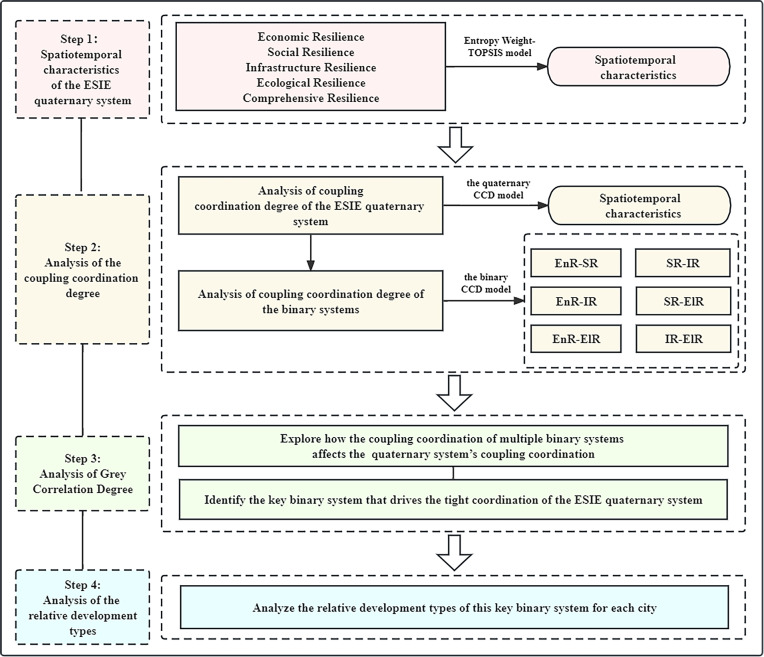
The theoretical framework of this study.

To sum up, this study systematically refines the strategies for improving urban resilience from a relatively novel perspective, aiming to provide scientific theoretical support and actionable insights for the improvement of urban resilience (Fig 2). First, the entropy weight-TOPSIS method is used to measure and analyze the urban comprehensive resilience (CR) and the four subsystems (EnR, SR, IR, ElR) in the study area. EnR, SR, IR, and ElR subsystems interact in pairs to form six binary systems (EnR-SR, EnR-IR, EnR-ElR, SR-IR, SR-ElR, IR-ElR). Furthermore, the ESIE quaternary system is a dynamic system formed by the interaction of the six binary systems, and its overall CCD must be affected by the CCD of binary systems [44]. Therefore, from a systems theory perspective, this paper deeply explores the coupling and coordination relationship of the ESIE quaternary system and six binary systems. The objective is to uncover the intrinsic mechanism of coupling and coordination that drives the development of urban resilience. The grey correlation model is a multi-factor statistical analysis technique and it can calculate the correlation degree between various factors and the system performance to quantify the impact of these factors on the overall performance of the system [45]. This study employs the grey correlation model to evaluate the impact of the CCD of each binary system on the overall CCD of the ESIE quaternary system, and takes into account the coupling coordination status of binary systems to identify the key binary system that promotes the development of the overall coordination. Furthermore, according to this binary system, this study classifies the relative development types of each city in order to identify the key problem points of urban development. In this way, cities can be guided to formulate differentiated sustainable development policies according to their own types of development, so as to most effectively enhance their urban resilience.

## 2 Data and methods

### 2.1 Study area and data sources

Zhejiang Province administratively comprises 11 prefecture-level cities, namely Hangzhou, Ningbo, Wenzhou, Shaoxing, Huzhou, Jiaxing, Jinhua, Quzhou, Zhoushan, Taizhou, and Lishui. Among them, Hangzhou and Ningbo are vice-provincial level cities. This study utilizes empirical analysis by collecting indicator data from 2014 to 2022 across the 11 cities in Zhejiang Province. The data are obtained from the “Zhejiang Statistics Yearbook,” “China Urban Statistical Yearbook,” “China Urban Construction Statistical Yearbook,” as well as statistical yearbooks and bulletins from each prefecture-level city. The method of obtaining is through the cnki statistical yearbook platform website and the official websites of prefecture-level city statistics bureaus. The data obtained are characterized by high quality, authority and reliability. Partial missing data are supplemented using linear interpolation method. Linear interpolation is a simple and effective method to estimate missing values based on adjacent data points [46]. It applies to the time series characteristics of our data and is able to keep the trend of the data to a certain extent. The data sources can be found in S1 Table. Given that the indicators are from various sources and there are positive and negative indicators, we employ the polarization standardization method to process the indicator data.

For negative indicators:


$$\begin{array}{c} r_{ij}=\frac{\max\left(x_{ij}\right)\;\;\;\;\;\;\;-\;\;\;\;\;\;\;x_{ij}}{\max\left(x_{ij}\right)-\text{min}\left(x_{ij}\right)} \end{array}$$ (1)

For positive indicators:


$$\begin{array}{c} r_{ij}=\frac{\;\;\;\;\;\;\;x_{ij}-\text{min}\left(x_{ij}\right)}{\max\left(x_{ij}\right)-\text{min}\left(x_{ij}\right)} \end{array}$$ (2)

Where $r_{ij}$ is the standardized value; $x_{ij}$ is the value of the jth indicator for the ith evaluation object.

### 2.2 Methods

#### 2.2.1 Entropy weight-TOPSIS model.

The TOPSIS (Technique for Order Preference by Similarity to Ideal Solution) method is an approach used to approximate ideal solutions in decision-making processes. Its fundamental concept views decision problems as a collection system of geometric points, constructs distances to positive and negative ideal points, and evaluates each alternative based on its distance from the negative ideal point and proximity to the positive ideal point, accurately reflecting the disparities among various alternatives [47–49]. The traditional TOPSIS model often relies on subjective expert opinions to determine indicator weights, potentially leading to interference in the final results. In order to avoid such a situation, the entropy weight method with strong objectivity is adopted in this study. According to the variation degree of each indicator, the entropy weight of each indicator is calculated by using information entropy, and then the weight of each indicator is modified by the entropy weight, so as to obtain more objective indicator weights [50,51]. In summary, this study establishes the entropy weight-TOPSIS model to separately evaluate CR, EnR, SR, IR, ElR of cities in Zhejiang Province from 2014 to 2022 separately.

#### 2.2.2 Coupling coordination degree model.

The CCD model is generally utilized to measure the strength of interaction and the degree of coordinated development between two or more systems or subsystems. Compared with other methods to study coupling effect, such as nonlinear dynamics model and double exponential model, the structure of the CCD model is simpler and operation is more convenient [52]. So it is widely applied in evaluating the coordination status of industries, economies, and regional development [53–57]. In this study, the CCD of the ESIE quaternary system across 11 cities in Zhejiang Province is measured using the quaternary CCD model. The CCD between any two subsystems is measured using the binary CCD model.

The quaternary CCD model [1,57–59] is as follows:

(1) Coupling degree formula

In the quaternary CCD model, coupling degree reflects the degree of interaction among subsystems of the ESIE quaternary system.


$$\begin{array}{c} C={\left[\frac{U_{1}U_{2}U_{3}U_{4}}{{\left(\frac{U_{1}+U_{2}+U_{3}+U_{4}}{4}\right)}^{4}}\right]}^{\frac{1}{4}} \end{array}$$ (3)

Where C is the coupling degree, and the value range is [0,1]. The larger the C value, the higher the coupling degree of the ESIE quaternary system. When C = 0, it means that the ESIE quaternary system is in an unrelated state and develops towards disorder. When C = 1, it means that the ESIE quaternary system achieves benign resonance coupling and tends to a new ordered structure. $U_{1}$, $U_{2}$, $U_{3}$ and $U_{4}$ are EnR, SR, IR and ElR, respectively.

(2) System comprehensive development index formula

$$\begin{array}{c} T=\frac{U_{1}+U_{2}+U_{3}+U_{4}}{4} \end{array}$$ (4)

Where T is the comprehensive development index of the four subsystems. This study considers that the four subsystems are equally important, so they are equally weighted.

(3) Coupling coordination degree formula

Coupling degree can only be used to describe the size of the coupling effect between more than two systems and it can’t reflect the development of the system is good or bad [60]. Therefore, it is necessary to use coupling coordination degree to measure the coupling coordination development of the system.


$$D=\sqrt{C\times T}$$ (5)

Where D is the coupling coordination degree, and the larger the D value is, the stronger the coupling coordination relationship of the ESIE quaternary system is.

The binary CCD model [57,60,61] is as follows:

(1) Coupling degree formula

In the binary CCD model, coupling degree reflects the degree of interaction between subsystems of a binary system.


$$\begin{array}{c} C={\left[\frac{U_{1}U_{2}}{{\left(\frac{U_{1}+U_{2}}{2}\right)}^{2}}\right]}^{\frac{1}{2}} \end{array}$$ (6)

Where C is the coupling degree and the value range is [0,1]. The larger the C value, the higher the coupling degree of the binary system. $U_{1}$ and $U_{2}$ are two subsystems of EnR, SR, IR and ElR, respectively.

(2) System comprehensive development index formula

$$\begin{array}{c} T=\frac{U_{1}+U_{2}}{2} \end{array}$$ (7)

Where T is the comprehensive development index of the two subsystems. This study considers that the two subsystems are equally important, so they are equally weighted.

(3) Coupling coordination degree formula

$$D=\sqrt{C\times T}$$ (8)

Where D is the coupling coordination degree, and the larger the D value is, the stronger the coupling coordination relationship of the binary system is.

#### 2.2.3 Grey correlation model.

The grey correlation model is primarily used to study matters where some information is known, but part of the information is unknown. Grey correlation analysis involves drawing sequence curves based on various factors and then judging the correlation degree based on the similarity of the geometric shapes of these sequence curves. The basic idea is to reflect the closeness of the correlation relationship based on the changing trends between the reference sequence and each comparative sequence, and it measures the relative strength of the influence of the comparative sequences on the reference sequence [45,62]. Binary systems are important components of the ESIE quaternary system, with some unclear information. Therefore, the grey correlation model is utilized in this study to analyze how the CCD of binary systems influences the CCD of the ESIE quaternary system. The higher the value of correlation degree, the greater the degree of influence.

The analysis process is as follows:

Step 1: Determine the reference sequence that reflects the characteristics of the system behavior (the CCD of the ESIE quaternary system) and the comparative sequences that affect the system behavior (the CCD of binary systems).

Step 2: Dimensionless processing. Since the dimensionless processing of the data has already been completed when calculating CCD, this step is omitted here.

Step 3: Calculate the association coefficient.


$$\begin{array}{c} \varepsilon_{i}\left(k\right)=\frac{\underset{i}{\min}\;\underset{k}{\min}\;|y\left(k\right)-x_{i}\left(k\right)|+\underset{i}{\;\;\;\rho \;\;\text{ max}}\;\underset{k}{\max}\;|y\left(k\right)-x_{i}\left(k\right)|}{\left|y\left(k\right)-x_{i}\left(k\right)\right|+\underset{i}{\;\;\;\rho \;\;\text{ max}}\;\underset{k}{\max}\;|y\left(k\right)-x_{i}\left(k\right)|} \end{array}$$ (9)

where $\varepsilon_{i}\left(k\right)$ is the association coefficient; y(k) is the reference sequence; $x_{i}\left(k\right)$ is a comparative sequence;  ρ  is the resolution coefficient and the value range is [0,1], usually 0.5.

Step 4: Calculate the correlation degree. For each binary system, calculate the mean value of its elements corresponding to the elements of the reference sequence to reflect the correlation between the CCD of each binary system and the CCD of the ESIE quaternary system. The closer the value is to 1, the higher the correlation. The formula is as follows:


$$\theta_{i}=\frac{1}{n}\underset{k=1}{\overset{n}{\sum }}\;\varepsilon_{i}\left(k\right)$$ (10)

where $\varepsilon_{i}\left(k\right)$ is the association coefficient; $\theta_{i}$ is the correlation degree.

### 2.3 Construction of indicator system

Based on the principles of scientific rigor, objectivity, comparability and quantification, this study refers to domestic and foreign literature on indicator system design and measurement [1,4,19,42,43,63–72], and adopts frequency statistics to screen out the indicators frequently used in empirical research. On this basis, combined with the theoretical framework of ESIE quaternary system and its multi-dimensional characteristics, a preliminary indicator system is constructed. According to the core attributes of indicators and their functions in the ESIE quaternary system, they are strictly divided into four subsystems: EnR, SR, IR and ElR, and further subdivided into multiple levels under each subsystem, so as to ensure the independence and logical clarity of indicators among different dimensions and avoid cross-dimensional repetition. The correlation analysis method is further used to test the independence of the indicators and eliminate the highly correlated indicators, so as to ensure the comprehensive information coverage of the indicator system and avoid redundancy. Finally, through multiple rounds of expert consultation and iterative modification, the evaluation indicator system as shown in Table 1 is constructed in this study. Each indicator is assigned a code according to its subsystem, categorized as follows: EnR (E1-E9), SR (S10-S17), IR (I18-I26), and ElR (E27-E37).

**Table 1:** Evaluation indicator system.

Subsystem	Dimension	Evaluation Indicators(Unit)	Indicator Nature	Indicator Weight	References
**Economic resilience**				**0.3147**	
	Economic Structure	E1-Proportion of tertiary industry in GDP (%)	+	0.0337	[42,43]
		E2-Proportion of primary industry in GDP (%)	-	0.0125	[43,65]
		E3-Number of industrial enterprises above designated size (units)	+	0.0401	[66]
	Economic Strength	E4-Per capita GDP (yuan)	+	0.0359	[42,63]
		E5-Per capita disposable income of urban residents (yuan)	+	0.0205	[42,63,65]
	Agricultural Production	E6-Crop area (thousand hectares)	-	0.0275	[67]
		E7-Grain production per capita (tons/10,000 people)	+	0.0422	[67,68]
	Economic Vitality	E8-Net export (100 million yuan)	+	0.0271	[1,68,69]
		E9-Number of wholesale and retail enterprises above quota (units)	+	0.0751	[67]
**Social resilience**				**0.3253**	
	Social Development	S10-Number of granted patent (items).	+	0.0586	[1,65]
		S11-Number of college students in 10,000 (people).	+	0.0650	[42,43]
		S12-Percentage of population under 18 & over 60 (%).	–	0.0157	[67,70]
		S13-Population density (people/km2).	–	0.0162	[63]
	Citizen Welfare	S14-The proportion of urban workers insured by basic medical insurance. (%)	+	0.0336	[1,66]
		S15-Proportion of people insured by unemployment insurance (%).	+	0.0433	[1,66]
	Social Services	S16-Public library holdings per capita. (volumes)	+	0.0264	[1,43,65]
		S17-Per capita fixed asset investment in urban municipal public facilities construction. (yuan)	+	0.0665	[42,43,68]
**Infrastructure resilience**				**0.2354**	
	Infrastructure Support	I18-Number of Internet broadband access users (10000 households)	+	0.0495	[65,71]
		I19-Urban road area per capita (m^2^)	+	0.0288	[43,65]
		I20-Number of buses per 10 000 people (vehicles)	+	0.0181	[43]
		I21-Number of doctors per 10,000 people (people)	+	0.0289	[19,64,69]
		I22-Number of beds in hospitals and health centers per 10,000 people (number)	+	0.0260	[42,43]
		I23-Number of hospitals per 10,000 people (pieces)	+	0.0312	[42,68]
		I24-Density of drainage pipeline in built-up area (km/km^2^)	+	0.0092	[63,72]
	Infrastructure Resources	I25-Annual electricity consumption (million KWH)	+	0.0402	[64,71]
		I26-Gas penetration rate (%)	+	0.0036	[63]
**Ecological resilience**				**0.1245**	
	Ecological Stress	E27-Industrial wastewater discharge per unit GDP (tons/10000 yuan)	–	0.0065	[42,65]
		E28-Industrial smoke (dust) emission per unit GDP (tons/billion yuan)	–	0.0036	
		E29-Industrial nitrogen oxide emissions per unit GDP (tons/billion yuan)	–	0.0095	
		E30-Industrial sulfur dioxide emission per unit GDP (tons/billion yuan)	–	0.0079	
		E31-Average annual concentration of fine particulate Matter (PM2.5) (μg/m^3^)	–	0.0155	[65]
	Ecological State	E32-Proportion of days with good air quality (%)	+	0.0156	[4,19]
		E33-Greening coverage rate of built-up area (%)	+	0.0256	[42,43]
		E34-Green space area of parks per capita (m^2^)	+	0.0277	[63,64,72]
	Ecological Management	E35-Comprehensive utilization rate of general industrial solid waste (%)	+	0.0038	[1,65]
		E36-Urban sewage treatment rate (%)	+	0.0065	[1,63,72]
		E37-Harmless disposal rate of domestic waste (%)	+	0.0023	[1,63]
					

Economic resilience is manifested as the stability of the economy in the face of uncertainties for a city [73], affecting the speed and quality of a city’s recovery after disasters. In this regard, nine indicators are selected to measure economic resilience from four aspects: economic structure, economic strength, agricultural production and economic vitality. At the level of economic structure, E1, E2 and E3 are selected to reflect the diversification of economic development. A larger proportion of the tertiary industry in the economic structure indicates a more reasonable industrial structure in the region [42,43], while a higher E2 may lead to increased vulnerability to disasters [43,65]. E3 measures the scale and concentration of the industrial sector, which often serves as a driving force for technological innovation and industrial upgrading. The more the number of industrial enterprises above designated size, the more obvious the supporting role of pillar industries, the more stable the economic structure, thus promoting high-quality economic development and enhancing economic resilience, which is a positive indicator [66]. E4 and E5 are important indicators for measuring economic strength. The higher their values, the greater the residents’ purchasing power and savings levels, which are conducive to coping with economic fluctuations, resisting disaster risks, and enhancing resilience [42,63,65]. At the level of agricultural production, crops are susceptible to disaster impacts, and a higher E6 value may actually weaken the ability to resist disasters [67]. E7 reflects a city’s food security situation [67,68]. Sufficient agricultural output can enhance the ability to withstand natural disasters and market fluctuations. In terms of economic vitality, E8 and E9 are selected to highlight the city’s international trade ability and market conditions [1,67–69]. High net export values and a thriving retail market imply a stronger adaptability of the economic system in the face of external shocks and internal changes.

Social resilience is manifested in the guarantee and potential for urban development that society possesses when a city encounters shocks, which affects the effectiveness of actions taken by the city in the face of disasters and the city’s ability to recover and grow post-disaster [74]. It focuses on the effectiveness of resource allocation and the sustainability of public services. This study selects eight indicators from three levels of social development, citizen welfare and social services to characterize social resilience. At the level of social development, S10 reflects the city’s scientific and technological innovation ability. The stronger the scientific and technological innovation ability, the more efficiently the city can use the advanced technology for disaster monitoring, early warning, emergency response and post-disaster recovery, so as to significantly improve the disaster resistance and recovery ability of the city [1,65]. S11 can effectively reflect the education level of the society. A higher education level means that more residents have the knowledge and skills of disaster prevention and reduction, and can take scientific and effective response measures when disasters occur, so as to reduce disaster losses and accelerate the post-disaster recovery process, which is a positive indicator [42,43]. S12 and S13 are demographic attributes, reflecting human capacity for disaster resistance and endurance [63,67,70]. At the level of citizen welfare, S14 and S15 are selected to reflect the importance and guarantee ability of the society to citizen welfare. A perfect social security system can provide residents with basic living security and enhance their coping ability and recovery potential in disasters, so they are positive indicators [1,66]. S16 and S17 are used to assess social service capacity, reflecting the level of resource allocation in the community to enhance public service capacity [1,42,43,65,68]. The higher the values, the more resources are invested in improving public service capacity, indicating a stronger overall service capability and a more significant ability to handle emergencies, thereby contributing to the enhancement of social resilience. Therefore, both indicators are positive.

Infrastructure resilience mainly focuses on hardware indicators and pays more attention to the scale, quality and operation status of existing facilities. It examines the infrastructure capabilities of a city in the event of a crisis, such as personnel evacuation, disaster response and external communication. It includes critical resources, networks, or services that a city depends on for survival, such as power, gas, telecommunications and transportation systems, as well as emergency service facilities like medical care and early warning [75]. This study selects the evaluation indicators of infrastructure resilience from two aspects: infrastructure support and infrastructure resources. At the level of infrastructure support, strengthening the planning and construction of infrastructure will help accelerate the construction of a modern urban system with scientific layout and complete functions, so as to enhance the urban basic support capacity and meet the growing needs of residents. I18 measures the ubiquity of digital infrastructure, contributing to the establishment of an effective disaster early warning system [65,71]. I19 and I20 demonstrate the capacity of infrastructure to support traffic flows. Larger per capita road areas and more public transport provide clear advantages for transport evacuation and emergency response during disasters [43,65]. I21, I22 and I23 together embody the ability to respond to and recover from risky disasters in terms of health care [19,42,43,64,68,69]. I24 reflects the guarantee level of urban municipal infrastructure, and the higher density of drainage pipeline in built-up area significantly enhances the resilience of cities to cope with flood disasters [63,72]. At the level of infrastructure resources, I25 and I26 reflect the construction level of resource-based infrastructure such as electricity and gas, providing essential resource guarantees for social development and human production and living activities [63,64,71].

Ecological environment is the spatial carrier of sustainable urban development. Ecological resilience is represented by the ability of a city to cope with ecological environment problems in the process of development and the self-adjustment ability of the ecological environment when confronted with shocks. 11 indicators are selected from the aspects of ecological stress, ecological state and ecological management. The indicators E27-E31 are used to reflect ecological stress [42,65]. Higher ecological stress indicates more severe ecological and environmental challenges faced by the city. Therefore, these indicators are all negative. At the ecological state level, E32-E34 reflect the current self-repair capacity and stability of the urban ecosystem. The higher the values of these indicators, the stronger the urban ecosystem’s adaptability to unforeseen risks and disasters, making them positive indicators [4,19,42,43,63,64,72]. At the ecological management level, E35-E37 represent the effective measures taken by urban governance to improve the ecological environment [1,63,65,72]. The implementation of these measures helps enhance the overall environmental quality of the city. Thus, these indicators are also positive, with higher values indicating better ecological management outcomes and stronger ecological resilience.

## 3 Results

### 3.1 Spatiotemporal characteristics of the ESIE quaternary system

**Fig 3:**
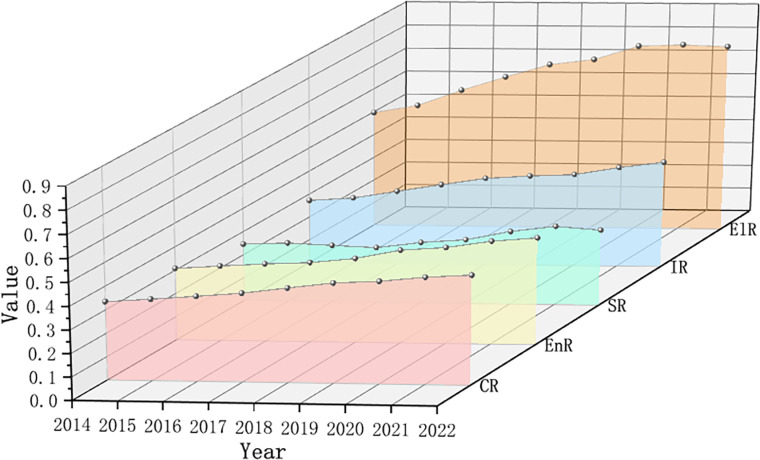
Temporal variation trend of urban resilience.

According to the temporal trend graph depicting the comprehensive resilience and the resilience of economic, social, infrastructure, and ecological dimensions in Zhejiang Province from 2014 to 2022 (Fig 3), the following observations can be made: (1) The development of economic resilience and infrastructure resilience has gradually synchronized over time: In 2014, the value of economic resilience was marginally higher than that of infrastructure resilience. However, by 2018, infrastructure resilience showed rapid improvement, reaching a steady development alongside economic resilience by 2022. The main reason is that Zhejiang province has actively responded to the national requirements of building high-quality cities and made great efforts to improve the ability of urban innovation governance in recent years. On the one hand, Zhejiang province has actively carried out industrial reconstructing, vigorously developed high-tech industry and modern service industry, promoting the steady improvement of economic resilience. On the other hand, cities abandon the original unreasonable and fragmented management methods, and constantly strengthen the management of infrastructure construction and urban risks [20]. The development of economic resilience and infrastructure resilience is also basically identical to the development level of comprehensive resilience, playing a mainstay role. (2) The development of social resilience lags behind, with a relatively slow growth rate in recent years and a small contribution to comprehensive resilience. The main reason is that there are different degrees of shortages or deficiencies in public health care, education and social security in urban Zhejiang Province. The lack of social resilience may restrict the further improvement of urban identity, cohesion, harmony and stability. (3) Ecological resilience is the highest among all subsystems and exhibited a relatively rapid growth rate. This is mainly due to the fact that Zhejiang Province actively explores urban construction models with Zhejiang characteristics, such as urban complexes, low-carbon cities, smart cities, etc., implementing green, low-carbon, and recyclable development practices, and commencing the construction of resource-conserving and environment-friendly cities with initial results. Specifically, during the statistical years, the greening coverage rate of built-up area and the green space area of parks per capita in Zhejiang Province maintained a high level, while pollution emissions were controlled at a low level, which improved ecological resilience.

**Fig 4:**
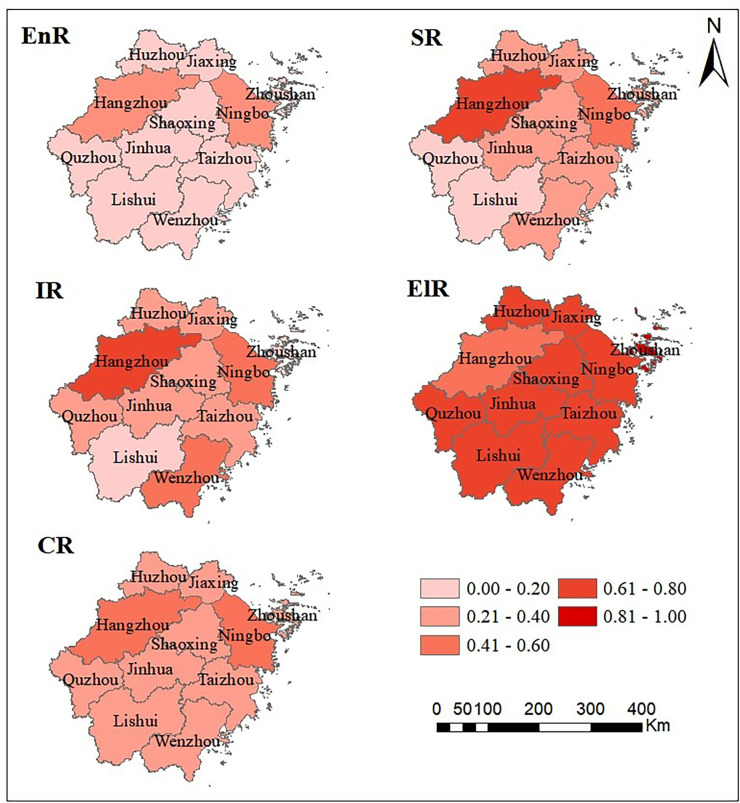
Spatial distribution pattern of urban resilience. Source of basemap: The open source map data service is provided by the National Platform for Common GeoSpatial Information Services (https://www.tianditu.gov.cn/), which meets the relevant provisions of the terms of service (https://www.tianditu.gov.cn/about/service).

Regarding urban resilience depicted by ArcGIS (Fig 4), Hangzhou and Ningbo, two economically developed cities, exhibit relatively high levels of social and infrastructure resilience compared to other cities, resulting in a significant disparity. This spatial pattern gives rise to a “Hangzhou-Ningbo dual-core driven” situation, which aligns with the spatial distribution of comprehensive resilience. However, the spatial distribution pattern of ecological resilience differs starkly: While Hangzhou leads in resilience across aforementioned dimensions, its ecological resilience remains at the lowest level in the entire province. Conversely, cities represented by Lishui and Quzhou have lower levels of development in economic, infrastructure, and social resilience, but correspondingly, these cities possess high levels of ecological resilience. The reasons for the development differences among the various resilience subsystems in cities within Zhejiang Province are closely related to their geographical locations, resource allocation, and regional development strategies. Hangzhou, as the capital of Zhejiang Province, has taken the lead in its development due to its resource tilt. Ningbo, by virtue of its port advantages, has developed strong foreign trade and port industries. In contrast, Lishui, Quzhou and other cities are located at the edge of the province, far from the provincial capital and the economic center, so they have relatively little resource allocation and relatively low level of economic development. However, these regions have unique advantages in ecological resources. The research results are in line with the reality, and to some extent reflect the contradiction between the regional ecological supply and the ecological demand of economic, social and infrastructure development caused by the industrialization of economic subsystem, the urbanization of social subsystem and the expansion of infrastructure subsystem [76,77].

Based on the above analysis, it can be observed that the urban resilience subsystems exhibit different development characteristics in terms of temporal evolution and spatial distribution: Temporally, EnR and IR develop in sync, while SR lags behind, and ElR is the highest among all subsystems. Spatially, the EnR, SR, and IR subsystems form a “Hangzhou-Ningbo dual-core driven” pattern, whereas ecological resilience has distinctly different distribution pattern. These differences reveal the unevenness and interdependence among the urban resilience subsystems in Zhejiang Province. Consequently, it is imperative to further analyze the coupled and coordinated development situation of the resilience subsystems.

### 3.2 Analysis of the coupling coordination degree

According to the assessment values of different subsystems of urban resilience from 2014 to 2022 in Zhejiang Province, the CCD model is utilized to calculate the CCD of the ESIE quaternary system and various binary systems of 11 cities analyzed. Investigation is conducted from temporal evolution and spatial distribution characteristics. For analytical convenience, this paper categorizes the value of CCD into the following six types based on relevant literature [42,78] as shown in Table 2.

**Table 2:** Classification of coupling coordination degree.

Type	Coupling coordination degree
Disorder	(0.0,0.5]
Bare coordination	(0.5,0.6]
Primary coordination	(0.6,0.7]
Moderate coordination	(0.7,0.8]
High coordination	(0.8,0.9]
Excellent coordination	(0.9,1.0]

#### 3.2.1 Analysis of coupling coordination degree of the ESIE quaternary system.

**Fig 5:**
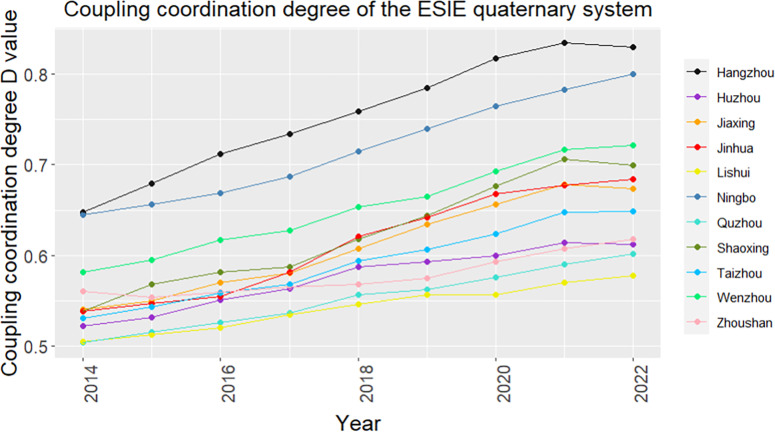
Temporal variation trend of CCD of the ESIE quaternary system.

From a temporal perspective, between 2014 and 2022, the CCD of the ESIE quaternary system in Zhejiang Province has increased steadily (Fig 5), with the level of CCD continuously improving. Except for Hangzhou and Ningbo, in 2014, the ESIE quaternary system of the other 9 cities were all in the state of “Bare coordination”. By 2018, while Hangzhou and Ningbo had reached “Moderate coordination”, Jiaxing, Shaoxing, Jinhua, and Wenzhou had reached “Primary coordination”, with the rest of the cities still at the level of “Bare coordination”. However, in the following three years, the quality of coupling coordination improved significantly. Apart from Lishui and Quzhou, all other cities have seen an upgrade in their CCD level, with Hangzhou leading the transition to “High coordination”. This progress is mainly attributed to Zhejiang Province’s active implementation of a development philosophy centered on innovation, coordination, green development, openness, and sharing in recent years, with a full commitment to promoting high-quality development. As a result, there has been positive progress in the coordinated development of urban economic, social, infrastructure, and ecological resilience.

**Fig 6:**
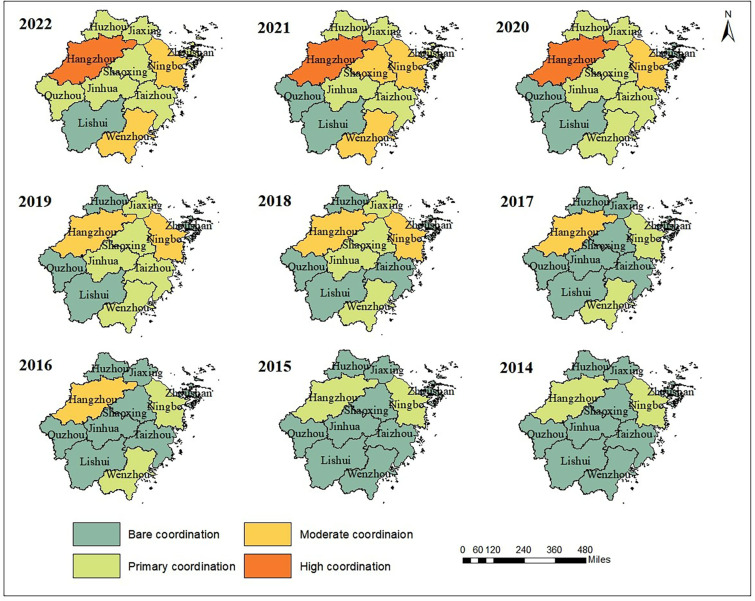
Spatial pattern evolution of CCD of the ESIE quaternary system. Source of basemap: The open source map data service is provided by the National Platform for Common GeoSpatial Information Services (https://www.tianditu.gov.cn/),which meets the relevant provisions of the terms of service (https://www.tianditu.gov.cn/about/service).

From a spatial perspective, the CCD of the ESIE quaternary system in Zhejiang Province generally exhibits characteristics of coordinated development among the cities in the linkage region, with Hangzhou and Ningbo as the core (Fig 6). The CCD of the ESIE quaternary system in Hangzhou and Ningbo starts at a relatively high level and shows a positive development momentum, continuously evolving towards higher levels of CCD. By 2022, Hangzhou had reached the status of “High coordination”, while Ningbo had reached the status of “Moderate coordination”. As the provincial capital, Hangzhou possesses certain advantages in policy formulation, cultural accumulation, and resource integration, making its level of CCD more prominent compared to Ningbo. The cities of Shaoxing, Jinhua, Jiaxing, and Taizhou, which are closer to Hangzhou and Ningbo, also exhibit higher levels of CCD. Proximity facilitates interaction among cities, including market access, industrial synergy, talent exchange, and resource sharing, all of which greatly contribute to enhancing economic resilience, social resilience, and infrastructure resilience. Additionally, neighboring cities can benefit from spillover effects from surrounding developed cities, which helps to improve their own urban resilience. Conversely, the inland cities of Lishui and Quzhou, located in mountainous areas, face complex terrain and are constrained by factors such as inconvenient transportation, uneven distribution of resources, and limited external communication. These conditions result in relatively lower CCDs of the ESIE quaternary system.

#### 3.2.2 Analysis of coupling coordination degree of binary systems.

**Fig 7:**
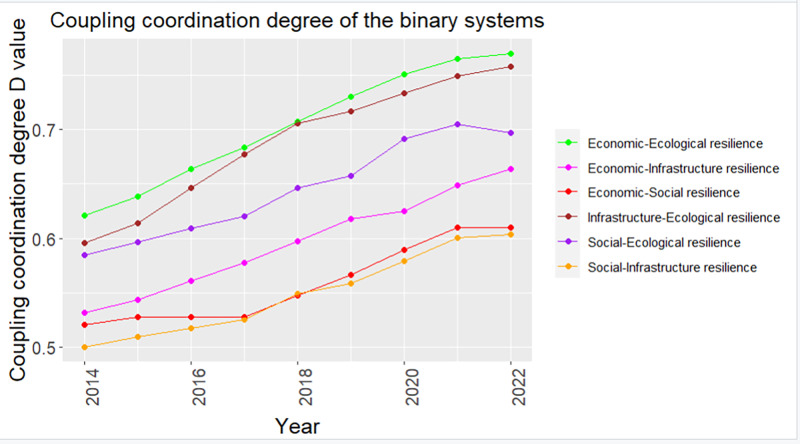
Temporal variation trend of CCD of binary systems.

**Fig 8:**
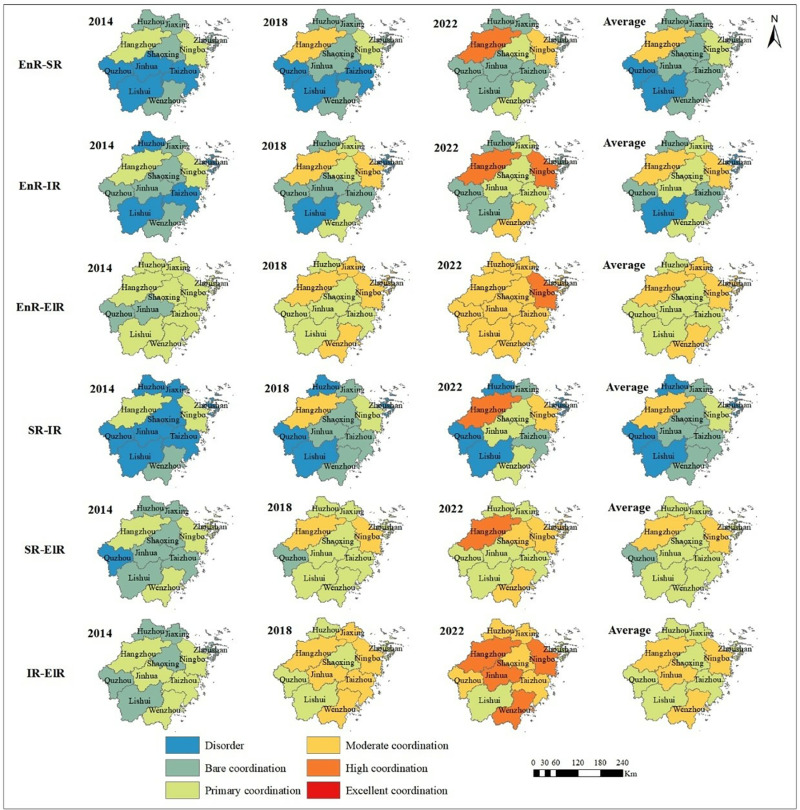
Spatial pattern evolution of CCD of binary systems. Source of basemap: The open source map data service is provided by the National Platform for Common GeoSpatial Information Services (https://www.tianditu.gov.cn/), which meets the relevant provisions of the terms of service (https://www.tianditu.gov.cn/about/service).

Figs 7 and 8 show the development of pairwise CCD between economic, social, infrastructure and ecological resilience subsystems in Zhejiang Province.

Temporally (Fig 7), the CCDs of the binary systems in cities of Zhejiang Province showed a consistently rising trend from 2014 to 2022, completing multi-level leaps in coupling coordination status. The EnR-ElR binary system has always been in the leading position. The IR-ElR binary system is second, and has a greater quality improvement. In 2014, the CCDs of binary systems were at the level of “Bare coordination” or even “Disorder”, with the highest reaching only “Primary coordination”, indicating a less coordinated development between the two subsystems. However, by 2022, the EnR-ElR and IR-ElR binary systems had basically reached “Moderate coordination”, and the SR-ElR binary system had also reached “Primary coordination”. This is highly associated with the implementation of ecological civilization construction policies in Zhejiang Province, the practice of sustainable development strategies, and the emphasis on the synchronous and continuous development of economy, society, infrastructure, and ecology [79]. Additionally, although the total levels of EnR-SR, EnR-IR, and SR-IR binary systems were not high, there had been some development during the statistical years. This indicates that although differences lie in the level of CCD among the various binary systems in Zhejiang Province, overall, they are all gradually improving.

In terms of spatial dimension (Fig 8), the CCD of the provincial capital Hangzhou and the eastern coastal city Ningbo is relatively high, reaching “Moderate coordination” or above in each binary system composed of economic, social, infrastructure, and ecological resilience. The southern coastal city of Wenzhou exhibits relatively high CCD levels in the EnR-IR and ElR-related binary systems, but there remains a significant gap in coupling coordination of EnR-SR and SR-IR binary systems compared to Hangzhou and Ningbo. In contrast, the development coordination degrees of other cities vary significantly, with CCDs in the SR-IR and EnR-SR binary systems predominantly at a “Bare coordination” level. This phenomenon suggests that population and resources in Zhejiang Province are flowing towards economically developed cities like Hangzhou, where economic growth drives progress in infrastructure and social development, thereby enhancing the CCD of various binary systems. In comparison, cities with slightly lower levels of economic development prioritize economic construction during their development process, paying relatively less attention to social and infrastructure aspects, resulting in obstacles to balanced development of coupling coordination. Addressing this uneven development trend requires targeted policy interventions and resource allocation to promote balanced development among cities within Zhejiang Province. As of 2022, Hangzhou and Ningbo have achieved an elevated level of CCD in all their binary systems, and the corresponding growth rates of CCD have also slowed down. However, other cities still face varying degrees of low CCD levels, with inland mountainous cities like Lishui having only achieved “Moderate coordination” in the EnR-ElR binary system, necessitating considerable attention in the province’s development planning. On the whole, the CCD of the binary systems in Zhejiang Province still shows the spatial distribution pattern of the leading development of Hangzhou and Ningbo during the study period. In the future, Zhejiang Province should focus on leveraging the radiating effect of cities like Hangzhou and Ningbo to enhance the development vitality of all cities and promote comprehensive coordinated development, especially among mountainous cities.

Comparing various binary systems reveals that CCDs are higher in the EnR-ElR, IR-ElR, and SR-ElR binary systems, all of which are closely related to ecological factors. This phenomenon indicates that ecological resilience is harmoniously developed with the other three subsystems, which is largely due to the superior performance of ecological resilience. Specifically, the introduction of relevant ecological policies and regulations in each city, the enhancement of public awareness of ecological protection and the greening of consumption concepts, the intensification of economic growth methods and the decarbonization of industrial structures, as well as the advancement and transformation of green science and technology, have contributed to the higher CCDs of eco-related binary systems in Zhejiang Province [4]. On the contrary, the CCD of the binary systems composed of economic resilience, infrastructure resilience and social resilience is low, indicating that the contradiction between economy, infrastructure and society in Zhejiang Province is prominent in recent years, which impedes the speed of urban development to a certain level. Resilience analysis indicates that social resilience performs comparatively poorly, and the regional development of social resilience and infrastructure resilience is unbalanced, making it difficult to form effective synergistic development of EnR-SR, EnR-IR, and SR-IR.

Analysis above reveals the dissimilarities in the coupling and coordination states of various binary systems in Zhejiang Province and also indicates the presence of poor coordination within the binary systems of the ESIE quaternary system. However, in order to find the optimal path to improve the CCD of the ESIE quaternary system, it is essential to continue delving into the core issues currently faced by the coupled and coordinated development of the ESIE quaternary system. This not only requires consideration of the existing CCD level of binary systems but also needs to be viewed along with the influence of the CCD of binary systems on the overall CCD. With the aim of identifying the key binary system that drives the tight coordination of the ESIE quaternary system, it is required to further analyze the specific impact of the CCD of each binary system on the overall CCD.

### 3.3 Analysis of grey correlation degree

The preceding text employs the CCD model to analyze both the overall CCD of the ESIE quaternary system and the pairwise CCD of each binary system. The ESIE quaternary system is a dynamic system formed by the interaction of multiple binary systems, and its overall CCD is inevitably influenced by the CCD of each binary system. Therefore, exploring the changes of overall CCD of the ESIE quaternary system brought about by CCD of multiple binary systems becomes an important pathway to clarify the internal operation mechanism of this collaborative system. This study employs the grey correlation model to systematically quantify the correlation between the CCD of each binary system and the overall CCD of the ESIE quaternary system. Through comparative analysis, it identifies which binary system has the greatest impact on the overall CCD.

**Fig 9:**
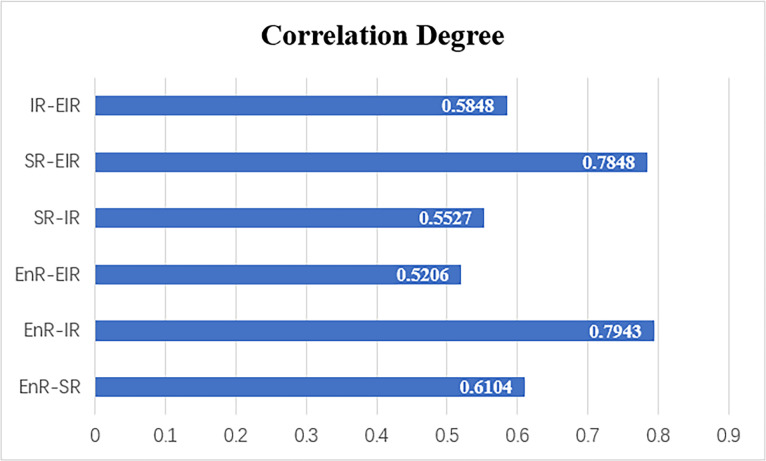
Grey correlation results.

The analysis results (Fig 9) indicate that the correlation degree spans from 0 to 1, where higher correlation degree, stronger correlations between the evaluation items and the overall CCD, suggesting a greater impact on overall CCD. Among the six evaluation items in this study, the EnR-IR binary system receives the highest rating (with a correlation degree of 0.7943), followed by the SR-ElR binary system (with a correlation degree of 0.7848). The lowest rating is for the EnR-ElR binary system (with a correlation degree of 0.5206).

To understand the core issue in the coupled and coordinated development of the ESIE quaternary system, it is vital to consider both the CCD level of binary systems and the magnitude of their effect upon the overall CCD. Combined with the previous analysis of the CCD of the binary systems, it is evident that the EnR-IR binary system has a lower level of CCD and exerts the dominant effect on the overall CCD. As for the SR-ElR binary system, although it also effects significantly on the overall CCD, its current level of CCD is relatively high. Similarly, although the SR-IR binary system currently has a lower level of CCD, its influence on the overall CCD is small. Therefore, these two binary systems do not require immediate priority. In summary, the coordinated development of the EnR-IR binary system is the foundation and key to the coordinated development of the ESIE quaternary system. It is necessary to further strengthen the coupling and coordination between these two subsystems from a policy perspective, thereby driving and enhancing the coupling and coordination of the ESIE quaternary system.

### 3.4 Analysis of the relative development types of the EnR-IR binary system

In order to delve into the developmental status of the EnR-IR binary system in Zhejiang Province, and to identify shortcomings in each city, this study compares the levels of economic resilience and infrastructure resilience of cities in 2022. It categorizes them into two development types: economic resilience lagging and infrastructure resilience lagging [61], as shown in Table 3, providing reference for formulating strategies to guide future urban development.

**Table 3:** Relative development types of EnR-IR binary system.

Relative Development Type	City
Economic resilience lagging	Hangzhou, Ningbo, Jiaxing, Jinhua, Taizhou, Wenzhou
Infrastructure resilience lagging	Huzhou, Shaoxing, Quzhou, Zhoushan, Lishui

As depicted in Table 3, six cities, namely Hangzhou, Ningbo, Jiaxing, Jinhua, Taizhou and Wenzhou, belong to the type of economic resilience lagging. Most of these cities are provincial capital or key development objects in the province. They have experienced rapid economic development and have also concentrated more public service resources, with relatively complete urban infrastructure construction. However, due to the poor rationality of industrial structure and other reasons, the growth rate of EnR is smaller than that of IR, and the poor economic support ability is the main reason leading to the low level of coordination. Such cities should promote the comprehensive innovation of economic resilience system, adhere to opening up and deepening reform, and actively explore new ways to promote urban economic growth. They should optimize the economic structure, cultivate high-tech industries, expand employment capacity, increase the income of urban and rural residents through multiple channels, firmly grasp the strategic point of expanding domestic demand, and stabilize talent resources through policy benefits.

Conversely, Huzhou, Shaoxing, Quzhou, Zhoushan, and Lishui belong to the type of infrastructure resilience lagging, and the growth rate of IR of this type is smaller than that of EnR. The poor supporting capacity of urban infrastructure is the main reason for the low coordination level. Such cities should give priority to improving infrastructure resilience, increase the development and construction of urban resources and infrastructure, and constantly improve the subsystem in urban development practice, so that it can truly become the basic guarantee for the sustainable development of these cities. Specific measures can include: elevating investment in infrastructure construction, focusing on the development of key areas such as transportation, communication, and water conservancy to improve urban operational efficiency and residents’ quality of life. Additionally, it is essential to elevate the level of public services by optimizing the distribution of public service resources in education, healthcare, culture, and other sectors to ensure synchronous improvements in service quality and efficiency.

## 4 Discussion

Based on the urban resilience system theory, this study deeply analyzes the coupling and coordination effects among the urban economic, social, infrastructure and ecological resilience subsystems in Zhejiang Province, and identifies the key binary system that promotes the overall CCD of the ESIE quaternary system. The results are basically consistent with the actual situation of urban resilience in Zhejiang Province, and can better reflect the coupling and coordination relationship among urban economic, social, infrastructure and ecological resilience. The indicator system constructed in this study can be applied to the research on the coupling and coordination relationships of the ESIE quaternary system in other urban agglomerations after appropriate adjustments. Although this study takes Zhejiang Province as a case, the proposed CCD framework of the ESIE quaternary system and the methodology for exploring the key binary system promoting urban coordinated development are universal. They can be extended and applied to other regions. By combining the actual development status of the local resilience system, this research offers theoretical guidance for the study of coupled and coordinated development of the ESIE quaternary system in other regions. At the same time, the model and method adopted by this study are scientific and stable, and have been widely used in the study of the coupling and coordination relationship of urban resilience subsystems [19,42,43,80]. The research methods and conclusions of this paper not only provide a scientific basis for the urban development strategy of Zhejiang Province but also offer a reference for the study of coupled and coordinated development of the ESIE quaternary system in other countries and regions.

The results show that the CCD of the ESIE quaternary system in Zhejiang Province continues to improve, showing a spatial pattern with Hangzhou and Ningbo as the dual core and the coordinated development of regional cities. This evolution process is in line with the high-quality development policy practice adopted by Zhejiang Province. At the same time, from the perspective of regional development strategy, Zhejiang Province proposed the spatial development idea of “three belts, three circles, one group and two districts”, which required to promote the formation of main functional zones and accelerate the construction of Hangzhou, Ningbo, Wenzhou urban economic circle and central Zhejiang city cluster [81]. This strategy effectively spawns the current spatial pattern of Zhejiang Province. The actual development situation aligns well with the findings of this study, confirming the reliability of the research results.

In the grey correlation analysis, the CCD of the EnR-IR binary system has the most significant influence on the CCD of the ESIE quaternary system. Relevant studies show that there is an interactive effect between regional economic development and infrastructure construction [82,83]. Infrastructure provides an indispensable hard environment for urban economic development and is the support and guarantee for the flow of various network elements between cities. The higher the level of economic development, there will be enough endowment supply to develop education, communication, transportation and other infrastructure. The good operation of these infrastructure will escort the operation of economic order, and ultimately promote the upgrading of industrial structure and sustained economic growth. This high correlation between infrastructure resilience and economic resilience will more easily lead to the lack of internal stability of the ESIE quaternary system, which will affect the stability of the overall coupling and coordination. According to the coupling coordination analysis of the EnR-IR binary system (Fig 8), it can be seen that almost all cities achieve hierarchical leap-over during the study period. The CCD of various cities is significantly different, with overall fluctuations being pronounced. Therefore, the fluctuation of the CCD of the EnR-IR binary system has a great impact on the overall CCD. In addition, the grey correlation results also show that the CCD of the EnR-ElR binary system has the least influence on the overall CCD. In the field of environment and economy, decoupling means that the link between economic growth and environmental pollution no longer exists [84]. GAI et al. [85] have found that the relationship between the indicators of resource and environmental pressure and economic growth in Zhejiang Province is mainly characterized by relative decoupling, indicating that the interaction effect between economy and ecology is relatively weak. This is a possible reason why the EnR-IR binary system a relatively small impact on the overall CCD. At the same time, the results of this study indicate that the ecological resilience and economic resilience in Zhejiang Province are relatively high. Besides, the CCD level of the EnR-ElR binary system has been in a high state, and the CCD level has a small change range during 2014–2022. This finding is consistent with the results of Yin et al. [86] and HONG et al. [87], which shows that the coordination relationship between EnR and ElR has been relatively mature and stable. Therefore, with the improvement of the CCD level of EnR-ElR binary system, its marginal contribution to the overall CCD will also show a decreasing trend. And its role in the overall CCD of the ESIE quaternary system is relatively weak.

Compared with the international research, the current focus of international scholars primarily lies in the evaluation of resilience and the construction of resilience mechanism [51,68,70], but there are few systematic discussions on the coupling and coordination relationship among various dimensions of urban resilience. Although these studies have shown significant application value in guiding practical engineering and improving urban resilience, the overall improvement of urban resilience as a complex system needs more comprehensive and systematic theoretical support. This study fully considers the interaction between the subsystems of urban resilience, and from the perspective of coordinated development, opens up a new path of urban sustainable development. The research provides improved methods and theoretical guidance for the construction of urban resilience and sustainable urban development on a global scale. It promotes the expansion of urban resilience research to a deeper and broader field.

However, there are still some limitations in the study, especially in the comprehensive consideration of urban resilience system, such as institutional and cultural resilience and other dimensions have not been fully explored. In order to respond to the development needs of resilient cities, the spatial and temporal scope of the research needs to be expanded in order to compare the coupling and coordination of urban agglomerations in different regions, different sizes and different stages of development. In addition, this study still has room for improvement in the determination of indicator weights, and future studies can improve the robustness and credibility of research results by designing diversified weight combinations.

## 5 Conclusions and policy recommendations

This study employs the entropy weight-TOPSlS method and the CCD model to analyze the resilience of four subsystems as well as the CCD of the ESIE quaternary system and binary systems. On this basis, the paper quantifies the influence of CCD of the binary systems on the overall CCD using grey correlation analysis and considering the current development level of coupling coordination, the key binary system that promotes the coordinated development of the ESIE quaternary system is determined. The main conclusions are as follows: (1) The resilience of various subsystems in Zhejiang Province exhibits differentiated evolutionary trends. Economic resilience and infrastructure resilience grow synchronously, while social resilience lags behind and ecological resilience performs the best among the subsystems. Spatially, Hangzhou and Ningbo exhibit higher economic, social, and infrastructure resilience, while Lishui and Quzhou stand out in terms of ecological resilience. (2) The spatiotemporal analysis of the CCD of the ESIE quaternary system indicates that over time, the coupling coordination of most cities shows a stable upward trend. Spatially, it presents a pattern of coordinated development centered on Hangzhou and Ningbo, interacting with surrounding cities. The spatiotemporal characteristics of the CCD of the six binary systems are similar to that of the ESIE quaternary system. However, in terms of relative relationships, binary systems related to ecological resilience exhibit higher level of CCD, while contradictions between pairs of subsystems such as economic, infrastructure, and social resilience are more pronounced, hindering urban development. (3) The results of grey correlation analysis show that EnR-IR has the greatest influence on the CCD of the ESIE quaternary system. Moreover, cities exhibit differentiated characteristics in terms of EnR-IR development types.

According to the research results, taking into account the differences in urban regional characteristics, resource endowments and development models, this study puts forward a series of practical policy recommendations:

(1) Cities vary in their performance in terms of economic resilience, social resilience, infrastructure resilience, and ecological resilience, with each having its own strengths and weaknesses. Therefore, in the process of promoting sustainable urban development, refined development strategies should be formulated based on the resilience characteristics of different cities. For cities like Zhoushan and Lishui, which have obvious advantages in ecological resilience, it is recommended to continue consolidating and nurturing ecological strengths. They should adhere to the concept of “green development and ecological prosperity for the people” to support the construction of economic, social, and infrastructure resilience. Specifically, a comprehensive tourism pattern can be formed through the construction of an interconnected network covering wetlands, woodlands, greenways and transportation. This network will support the development of new tourism formats, such as characteristic homestays and ecological agriculture. The objective is to enhance the value of cultural landscapes and promote economic development. At the same time, strengthening the construction of eco-tourism service facilities can significantly improve infrastructure resilience, thus enhancing urban resilience in all aspects. For cities like Hangzhou and Ningbo, which have stronger economic resilience, emphasis should be placed on enhancing ecological resilience and emphasizing the harmonious coexistence between humanity and nature. Specifically, it is necessary to s promote the integration of mountains and rivers with urban and rural areas, the fusion of nature and culture, and the integration of development and protection. Moreover, it is important to guide the appropriate population agglomeration and the sound industrial development according to local conditions. This will effectively promote the optimal allocation of production factors and the reasonable flow across regions.
(2) The CCD level of the ESIE quaternary system varies among cities in Zhejiang Province. Therefore, in the process of promoting the construction of urban resilience, cities should actively break time and space restrictions, regional boundaries as well as administrative barriers, and coordinate the planning and design of urban sustainable development programs. An urban agglomeration structure with complementary functions can be built through the construction of multi-party collaborative governance network system across departments and levels. This approach will alleviate issues such as unbalanced and inadequate development, resource mismatch, and structural imbalance in the urban agglomeration of Zhejiang Province. To be specific, it is necessary to strengthen the radiation leading role of cities such as Hangzhou and Ningbo, which perform well in the CCD of the ESIE quaternary system. While developing the economic strength and extroverted service capacity, it is also necessary to strengthen the cooperation between these core cities and neighboring cities in industrial upgrading, export trade, foreign exchanges and infrastructure construction, so as to enhance the spillover effect on neighboring cities. At the same time, we should pay attention to the cultivation and development of those cities that have medium performance but obvious advantages in the CCD of the ESIE quaternary system, such as Wenzhou, Shaoxing, Jinhua and Taizhou, so that they become the key convergence points of regional development. For the cities with lower CCD of the ESIE quaternary system, more support should be given to help them achieve leapfrog development. For example, Huzhou can take the lead of the government to actively introduce advanced technologies and talents from the core circle of the Yangtze River Delta. By doing so, it can accelerate industrial upgrading and develop its local advantageous industries. Southwest inland cities such as Quzhou and Lishui can strengthen the transfer of government finance, build and improve infrastructure construction, and strengthen the connection between cities inside and outside the region. Through these measures, the construction of network development structure of urban agglomeration in Zhejiang Province can be finally promoted.
(3) Considering that the EnR-IR binary system is crucial to the CCD of the ESIE quaternary system, it is essential to prioritize the deep integration of economy and infrastructure. This involves strengthening the construction of provincial infrastructure and promoting interconnectivity between cities to break through geographical boundaries and neighborhood effects that constrain regional mobility. At the same time, it is also necessary to move beyond the “each-for-themselves” approach and eliminate administrative boundaries to exchange information on major economic decisions. This will facilitate the free flow of various factors within the region and promote coordinated regional economic development. Cities with economic resilience lagging should focus their development on promoting high-quality economic growth and maintaining synchronous growth and coordinated development of economic resilience and infrastructure resilience, so as to enhance the overall CCD of the ESIE quaternary system. These cities should specifically refine their industrial layout. For example, Hangzhou should rely on its first-mover advantage in digital economy to deepen the research and application of cutting-edge technologies such as artificial intelligence, big data and cloud computing. The city needs to build a digital industrial ecosystem, and promote the integration and innovation of information technology and traditional industries. Ningbo needs to focus on the comprehensive efficiency of the port economy, while accelerating the cluster development of advanced manufacturing, especially in the field of high-end equipment manufacturing, new materials and new energy to achieve technological breakthroughs. Wenzhou is committed to the transformation and upgrading of the private economy, and promotes the evolution of the traditional manufacturing industry to the direction of high value-added through technological innovation and brand building. For cities with infrastructure resilience lagging, they should base on their own development reality, scientifically allocate the scale of infrastructure investment and construction to avoid disorderly investment and construction duplication. For example, Lishui and Quzhou, situated in the mountainous southwestern region of Zhejiang Province, should strengthen the construction of transportation infrastructure in mountainous areas, especially the optimization of public transportation, as well as the improvement of eco-tourism facilities, so as to promote regional accessibility and sustainable development of eco-tourism. With its unique island location, Zhoushan should focus on the development of infrastructure related to Marine economy, such as the construction of island tourism facilities and Marine science and technology parks, in order to strengthen its function as a hub of Marine economy.

## Supporting information

S1 Table Raw data for measuring urban resilience. This includes raw data of urban resilience evaluation indicators of 11 prefecture-level cities in Zhejiang Province from 2014 to 2022. (XLSX)
